# Screening drug-target interactions with positive-unlabeled learning

**DOI:** 10.1038/s41598-017-08079-7

**Published:** 2017-08-14

**Authors:** Lihong Peng, Wen Zhu, Bo Liao, Yu Duan, Min Chen, Yi Chen, Jialiang Yang

**Affiliations:** 1 Key Laboratory for Embedded and Network Computing of Hunan Province, College of Information Science and Engineering, Hunan University, Changsha Hunan, 410082 China; 2 College of Information Engineering, Changsha Medical University, Changsha Hunan, 410219 China; 3 Hunan University of Commerce, Changsha Hunan, 410205 China; 4 College of Drug, Changsha Medical University, Changsha Hunan, 410219 China; 5 Department of Genetics and Genomic Sciences, Icahn School of Medicine, Mount Sinai, NY 10029 USA

## Abstract

Identifying drug-target interaction (DTI) candidates is crucial for drug repositioning. However, usually only positive DTIs are deposited in known databases, which challenges computational methods to predict novel DTIs due to the lack of negative samples. To overcome this dilemma, researchers usually randomly select negative samples from unlabeled drug-target pairs, which introduces a lot of false-positives. In this study, a negative sample extraction method named NDTISE is first developed to screen strong negative DTI examples based on positive-unlabeled learning. A novel DTI screening framework, PUDTI, is then designed to infer new drug repositioning candidates by integrating NDTISE, probabilities that remaining ambiguous samples belong to the positive and negative classes, and an SVM-based optimization model. We investigated the effectiveness of NDTISE on a DTI data provided by NCPIS. NDTISE is much better than random selection and slightly outperforms NCPIS. We then compared PUDTI with 6 state-of-the-art methods on 4 classes of DTI datasets from human enzymes, ion channels, GPCRs and nuclear receptors. PUDTI achieved the highest AUC among the 7 methods on all 4 datasets. Finally, we validated a few top predicted DTIs through mining independent drug databases and literatures. In conclusion, PUDTI provides an effective pre-filtering method for new drug design.

## Introduction

Identifying drug-target interaction (DTI) candidates is important in modern drug discovery^1–3^. Efficiently predicting possible DTIs helps accelerate research efforts in discovering multitarget drugs or multidrug targets^4, 5^. High-throughput screening provides more opportunities for exploring DTIs^3^. However, existing data about DTIs are still very limited. For example, although an estimated 35 million compounds exist in the PubChem database, only <7000 drug compounds have available association information on their corresponding targets^3^. Experimental determination of DTIs remains labor-intensive, time consuming, and limited to small-scale identifications^4, 6^. Therefore, appropriate computational methods are needed to screen DTI candidates to save time and cost of biomedical experiments^3^.

Traditional computational methods to predict DTIs can be divided into ligand-based methods^7^ and molecule docking methods^8^. Ligand-based methods^7^ might be limited when target proteins have no known association information^9^, while molecular docking methods^8^ are computationally costly and depend largely on the 3D structures of target proteins^3, 9^. To overcome these problems, multiple computational models have been increasingly exploited to determine potential DTIs^10–12^. These computational methods are generally classified into two main classes: network-based inference methods and machine learning-based prediction methods^3^. Network-based inference methods, such as multiple target optimal intervention model^13^, drug side-effect similarity-based inference model^14^, and random walk-based prediction model with restart on the heterogeneous network^10^, can be used to investigate novel DTIs even if the 3D structures of proteins are unknown. However, this kind of method cannot detect possible DTIs when drug-target pairs are unreachable in a DTI network^3^.

An increasing number of machine learning-based methods have been proposed for inferring DTI candidates among which supervised learning methods are the most widely used^3, 15^ because they have excellent predictive capability^3, 16^. For example, a kernel regression-based approach^17^ was proposed to predict possible DTIs from human enzymes, ion channels, GPCRs and nuclear receptors by integrating the chemical structures of drug compounds, sequence information of target proteins and known DTI networks into a unified framework. A supervised learning method^18^ based on a bipartite local model performs well, but it cannot predict DTI candidates for new drugs or targets^19^. A Regularized Least Square-based method^20^ defined Gaussian interaction profile kernel and Kronecker product kernel (Kron) to identify possible DTIs (*RLS*_*Avg*_ and *RLS*_*Kron*_). Kerneled Bayesian matrix factorization methods based on classification and regression^21^ obtained good predictive performances (KBMF2K-classification and KBMF2K-regression). A contrastive divergence method^22^ combing restricted Boltzmann machines was developed to find DTI candidates. However, this method only utilized known DTI networks and did not take advantage of drug and target similarity networks^3^. A Random Forest (RF)-based learning approach^23^ was exploited to predict DTIs by integrating substructures of compounds, physicochemical and biomedical properties of proteins and known DTI networks. However, this approach cannot detect possible DTIs for a new drug or target without association information. To solve this problem, multiscale feature representation approach^24^ based on deep learning, random projection ensemble method^25^ and support vector machine (SVM)^12^ were utilized to infer DTI candidates for new drugs or targets.

Supervised learning have demonstrated satisfactory classification capability^15^. However, their classification accuracy and robustness depend on the training dataset, wherein negative and positive samples are equally important. For potential DTI identification, unfortunately, positive samples (known DTIs) are rare, and experimentally validated negative samples (non-interacting drug-target pairs) are difficult to achieve or even unavailable^26, 27^. Thus, supervised learning-based models can only randomly generate negative samples from unlabeled drug-target pairs^26, 27^. However, these unlabeled datasets possibly include both positive and negative DTIs^28^. Thus, this inaccurate method for negative sample selection severely disturbs generation capability of the models and result in overoptimistic classification results^3, 9, 26^. Therefore, it is highlighted in refs 3 and 9 that extracting highly credible Negative DTI Samples (NDTISs) is one of the important developments in predicting DTIs.

It is assumed in ref. 26 that the compounds dissimilar to every known drug are not much likely to associate with proteins that interact with the known drugs, and vice versa. Based on the assumption, a systematic method, NCPIS, is presented to build up a set of reliable negative DTI samples. Reference 28 treated unknown DTIs as unlabeled samples and used three methods (KNN, random walk with restarts and heat kernel diffusion) to extract reliable negative examples and likely negative examples based on PU learning and target similarity information.

Positive and unlabeled (PU) learning^29–31^ has been widely applied to classify unlabeled data. The techniques can be categorized into two main classes based on different strategies that deal with unlabeled samples^29, 31^. One group of methods simply extract reliable negative samples from the unlabeled data and learn a classifier using positive and reliable negative data. The Spy-EM^32^ and Roc-SVM^33^ are two representative techniques. The Spy-EM method^32^ classified unlabeled texts based on a naive Bayesian classifier and an expectation maximization (EM) algorithm. The Roc-SVM method^33^ classified unknown documents by integrating the Rocchio technique and SVM. However, only known positive samples and extracted negative samples are available, and ambiguous samples (remaining unlabeled samples) are excluded in these two methods, thereby limiting their performances^29^.

Another group of methods fully utilized the ambiguous samples, except for positive and reliable negative data, during the learning process^29–31^. Micro cluster-based PU learning method (LELC)^30^ was applied to select high-quality negative samples and likely positive and negative samples from the unlabeled samples for data stream classification. LELC algorithm^30^ obtained more robustness than existing data stream classification techniques. However, LELC method absolutely imposed samples of the whole micro-cluster on either class^29, 31^. Therefore, misclassification may be generated when parts of the samples are close to the positive class, and the other samples are more biased toward the negative class in a micro-cluster^29^. To solve this problem, a similarity-based PU learning technique (SPUL)^29^ extended the standard SVM to explicitly identify the ambiguous examples. PU learning approach mixing population and individual properties (MPIPUL)^31^ detected deceptive reviews by mixing global and local information. Both techniques took full advantage of the similarities between samples for the easily misclassified ones, therefore, they obtained significantly higher improvement than the LELC algorithm.

Considering PU learning-based methods and various biological information related to drugs and targets, we first developed a Negative DTI Samples Extraction method, NDTISE, to screen strong negative DTI examples. A novel DTI screening framework, PUDTI, was then designed to infer new drug repositioning candidates of existing drugs and targets by integrating NDTISE, probabilities that the ambiguous samples belong to the positive and negative classes, and an SVM-based optimization model.

## Results

**Figure 1:**
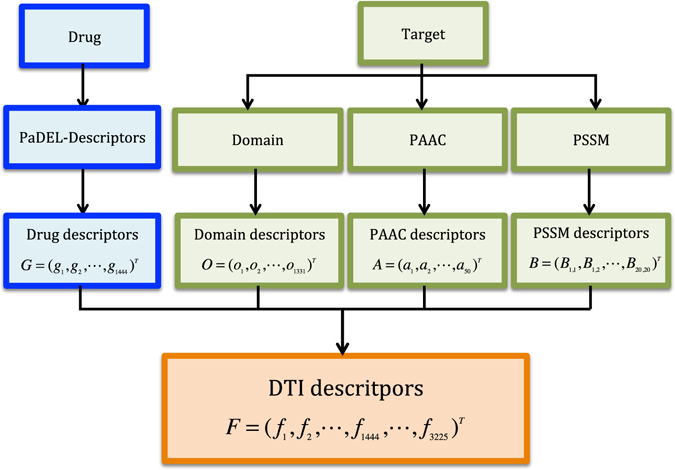
Representing each DTI as a vector.

**Figure 2:**
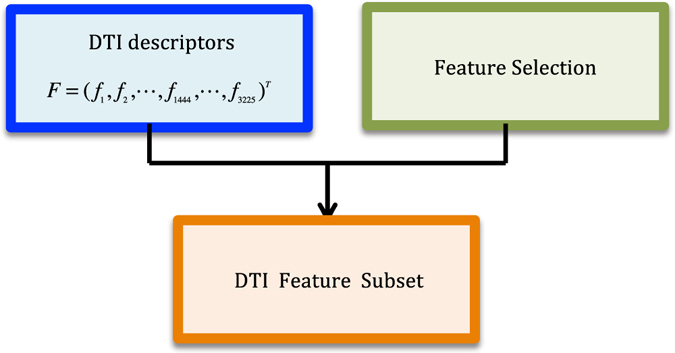
Selecting feature subset of DTIs.

**Figure 3:**
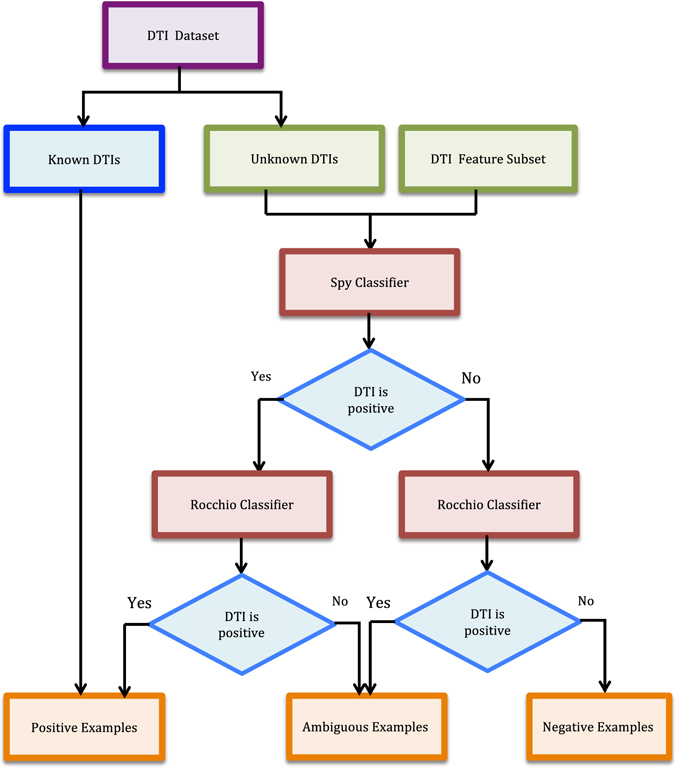
The NDTISE method.

**Figure 4:**
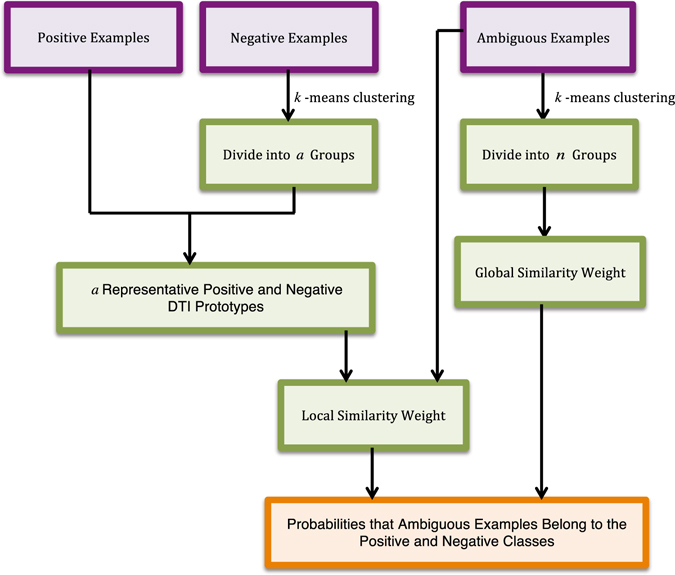
Computing the similarity weights of remaining ambiguous examples.

**Figure 5:**
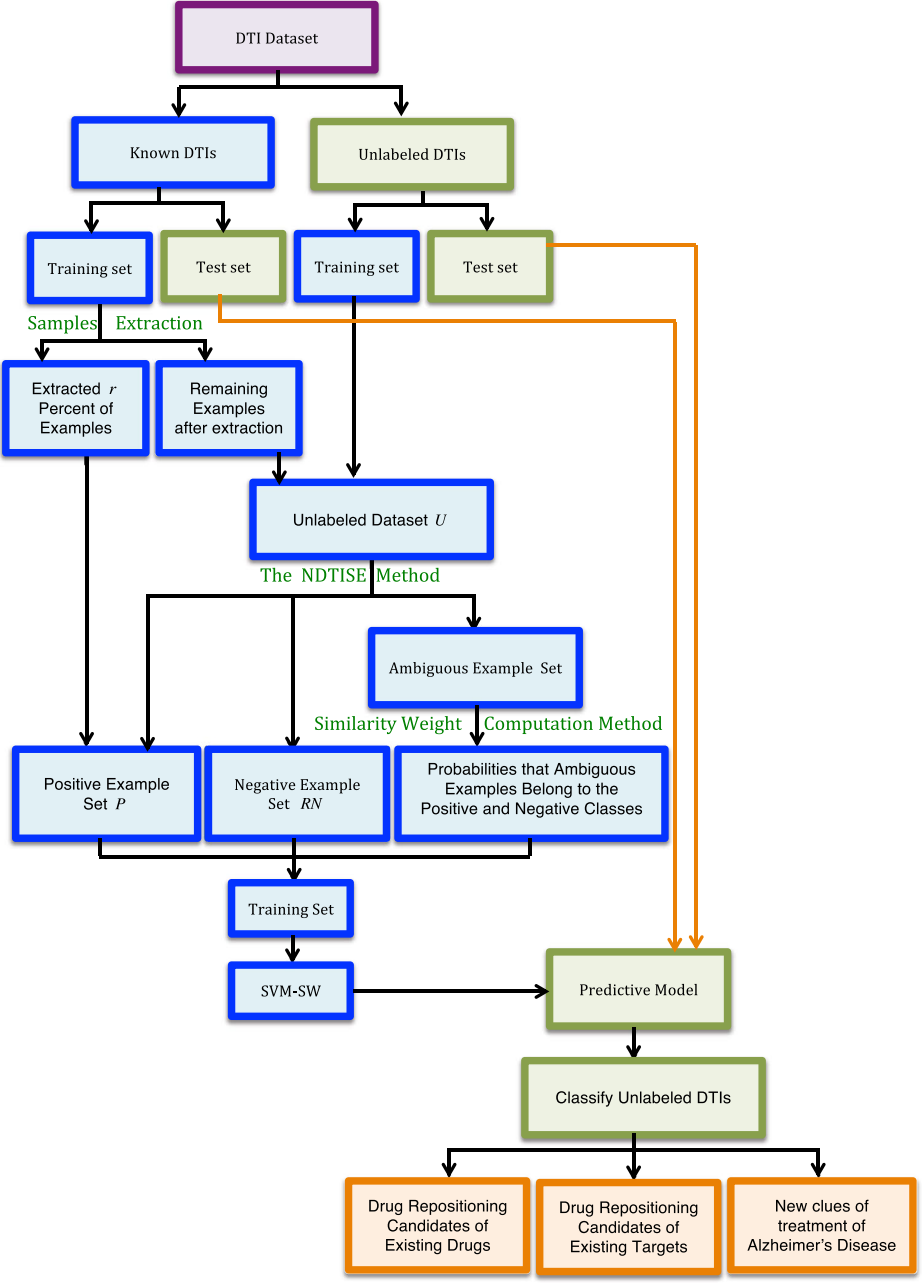
Classify unknown DTIs based on SVM-SM.

Our goal is to (a) improve DTI predictive accuracy based on the PUDTI framework; (b) effectively identify drug repositioning candidates for existing drugs and targets; (c) provide new clues of the treatment for Alzheimer’s diseases. The central idea is to extract NDTISs based on PU learning. Figures 1, 2, 3, 4 and 5 show the illustration of the PUDTI framework. The framework consists of five main parts: representing each DTI as a vector based on various biological information, selecting feature subsets of DTIs, constructing strong NDTISs, computing the similarity weights of the ambiguous examples, and building an SVM-based optimization model.

We evaluated whether our proposed PUDTI framework can identify potential DTIs properly. We presented extensive experiments under different experimental settings. (1) We compared the performances of our proposed NDTISE method with random selection method and NCPIS on a DTI data provided by NCPIS^26^. (2) We evaluated our proposed PUDTI framework on four classes of datasets from human enzymes, ion channels, GPCRs and nuclear receptors, respectively. (3) We compared the performances of 5 representative DTI prediction models including BLM, RLS-Avg, RLS-Kron, KBMF2K-classification and KBMF2K-regression by applying the negative samples predicted by NDTISE, random selection and NCPIS, respectively on the DrugBank data. (4) Parts of new drug repositioning candidates of existing drugs and targets are identified. (5) New clues of the treatment of Alzheimer’s disease are inferred.

We executed the feature selection method and ranked each feature based on their discriminant capability scores in constructed positive sample set *P* and unlabeled sample set *U*. Moreover, we screened the top 300 features for DTIs. Considering previous studies^25^ and our test, we chose the radial basis kernel as the kernel function because of its good boundary response^24^. The parameters *C*_1_, *C*_2_, *C*_3_ and *C*_4_ were set with a step size of 2^−4^ in the range [2^−5^, 2^5^].

### Performance Comparison of Different Negative Sample Selection Methods

We compared three different negative sample selection methods including NDTISE, random selection and NCPIS on the DTI data provided in the paper^26^ using six classical classification models including naive Bayes (NB), *k*-nearest neighbor (*k*NN), L1-logistic (L1-R) and L2-logistic regression(L2-R), RF and SVM. The parameters on these classifiers were set as the default values provided by ref. 26. The negative ratio in NCPIS was chosen as 3. The *k* for *k*NN algorithm was set as 1. Both the codes of the Spy and Rocchio classifiers^32, 33^ can be achieved from the LPU system^30^ (http://www.cs.uic.edu/liub/LPU/LPU-download.html).

A total of 10 trials of pairwise 5-fold cross-validation^9, 26^ were used to measure the NDTISE method against random selection method and NCPIS. (1) The drug-target pairs *D* (interacting or non-interacting) in the gold standard dataset were randomly partitioned into five mutually exclusive subsets that were roughly equal in size $\{{D}_{1},{D}_{2},\ldots ,{D}_{5}\}$. (2) In each round $t\in \mathrm{\{1,}\,\mathrm{2,}\ldots ,\,\mathrm{5\}}$, one drug-target pair set *D*_*t*_ was regarded as a test set, and the entries in *D*_*t*_ were masked. The remaining four subsets *D*\*D*_*t*_ were taken as training sets to recover the masked true labels in *D*_*t*_. (3) The experiment was repeated 10 times to avoid sampling bias, and the average predictive performance over the 5-folds for 10 trials was used as the final result.

To extract sub-datasets for PU learning, we specially conducted the following setting: we randomly extracted *r* percent of samples from known DTI dataset in the training set to form a positive sample set *P*. The remaining samples from the known DTI dataset and unknown drug-target pairs in the training set were used together to form an unlabeled dataset *U*. We firstly set *r* = 10, and evaluated the performances of the NDTISE method by increasing *r*. We observed that the NDTISE method is basically stable when *r* is no less than 30. Therefore, we set *r* as 30 in this study. The above six classifiers utilized *P* and *RN* extracted by the three negative sample selection methods as positive and negative samples, respectively. SVM-SW computed the similarity weights of the ambiguous samples besides *P* and *RN*.

We listed in Table 1 the performances of the three negative sample selection methods using respective classification models in terms of precision, recall, f-measure and AUC. NDTISE outperforms the other two methods in 4 classification methods and achieves comparable performances to NCPIS in the other two classification methods. Compared to random selection method, for instance, the average AUC values on NDTISE increased by 17.29%, 36.10%, 5.89%, 7.03%, 26.79% and 25.08% in NB, *k*NN, L1-R and L2-R, RF and SVM, respectively. The F-measure values on NDTISE also increase by 29.34%, 55.38%, 15.60%, 15.54%, 53.31% and 58.60% from naive Bayes to SVM. Compared with NCPIS, NDTISE was found to be superior in NB, *k*NN, L1-R and L2-R. For instance, the AUC values of NDTISE increased by 10.64%, 2.59%, 1.82% and 2.02% from NB to L2-R. Moreover, the F-measure values of NDTISE increased by 12.87%, 3.88%, 4.69%, and 3.08%. The observations indicated that NDTISE can effectively screen negative DTI samples.

**Table 1:** Performance comparison of six classical classification models on random selection method, NCPIS and NDTISE.

Metric	Negative DTIs	NB	kNN	L1-R	L2-R	RF	SVM
Precision	Random	0.338	0.458	0.786	0.787	0.529	0.700
	NCPIS	0.361	0.716	0.823	0.837	**0**.**847**	**0**.**969**
	NDTISE	**0**.**422**	**0**.**759**	**0**.**877**	0.**842**	0.840	0.965
Recall	Random	0.376	0.306	0.622	0.631	0.306	0.261
	NCPIS	0.560	0.882	0.749	0.773	0.**824**	0.**883**
	NDTISE	0.**625**	**0**.**897**	0.**775**	**0**.**817**	0.821	0.876
F-measure	Random	0.356	0.367	0.694	0.700	0.388	0.380
	NCPIS	0.439	0.790	0.784	0.804	0.**835**	0.**924**
	NDTISE	**0**.**504**	**0**.**822**	**0**.**823**	**0**.**829**	0.830	0.918
AUC	Random	0.622	0.593	0.879	0.873	0.694	0.705
	NCPIS	0.672	0.904	0.917	0.920	0.**954**	0.**942**
	NDTISE	**0**.**752**	**0**.**928**	**0**.**934**	**0**.**939**	0.948	0.941

Although the performances of NDTISE were slightly lower than NCPIS in the RF and SVM, our proposed PUDTI framework based on the SVM-SW classifier was better than NCPIS, as shown in Table 2. The results indicated that considering the probabilities that the ambiguous samples belong to the positive and negative classes may help improve classification performance.

**Table 2:** Performance comparison on SVM and SVM-SW.

Metric	Precision	Recall	F-measure	AUC
SVM	0.965	0.876	0.918	0.941
SVM-SW	**0**.**973**	**0**.**892**	**0**.**931**	**0**.**962**

### Comparison on Four Classes of Datasets Provided by Yamanishi *et al*

Yamanishi *et al*.^17^ screened 90, 635, 1476 and 2926 interactions based on 54, 223, 210 and 445 drugs and 26, 95, 204 and 664 proteins from human nuclear receptors, GPCRs, ion channels and enzymes, respectively. Table 3 described the details. To demonstrate the performance of our proposed PUDTI framework, we compared it with 6 state-of-the-art methods on these four datasets: DBSI^11^, NetLapRLS^34^, KBMF2K^21^, NetCBP^27^, WNN-GIP^35^ and PUDT-Lan^28^. The six methods were used to predict potential DTIs from human nuclear receptors, GPCRs, ion channels and enzymes and the last method inferred possible DTIs based on PU learning.

**Table 3:** Datasets from human nuclear receptors, GPCRs, ion channels and enzymes^17^.

Dataset	Nuclear receptors	GPCRs	Ion channels	Enzymes
drugs	54	223	210	445
targets	26	95	204	664
interactions	90	635	1476	2926

We listed in Table 4 the average AUC values of these six methods and our proposed PUDTI framework. It is clear that PU-based prediction methods significantly outperform other methods on all four datasets, which suggests that extracting negative DTI samples from unlabeled drug-target pairs may help improve prediction performance. In addition, our proposed PUDTI framework is better than the PUDT-Lan method, which might due to the fact that we considered the probabilities that the ambiguous samples belong to the positive and negative classes in PUDTI.

**Table 4:** The average AUC values of different DTI prediction methods on four datasets.

Dataset	DBSI	NetLapRLS	KBMF2K	NetCBP	WNN-GIP	PUDT-Lan	PUDTI
Nuclear receptor	0.759	0.761	0.810	0.838	0.839	0.885	**0**.**907**
GPCR	0.803	0.826	0.840	0.823	0.872	0.878	**0**.**894**
Ion channel	0.803	0.793	0.802	0.803	0.775	0.831	**0**.**875**
Enzyme	0.806	0.802	0.812	0.825	0.861	0.884	**0**.**898**

### Comparison with Representative DTI Prediction Methods on the DrugBank data

We compared the performances of 5 representative DTI prediction models including BLM, RLS-Avg, RLS-Kron, KBMF2K-classification and KBMF2K-regression by applying the negative samples predicted by NDTISE, random selection and NCPIS, respectively on the DrugBank data. These methods were originally used to identify potential DTIs from human enzymes, ion channels, GPCRs and nuclear receptors, which were provided by ref. 17. For RLS-Avg and RLS-Kron, we set the parameters as (0.5, 0.5) and (0.5, 0.5), wherein the two classifiers obtained better classification performances than (1, 1) and (1, 1)^26^. We extracted strong NDTISs based on algorithm 1. The drug and protein similarity matrices can be calculated according to cosine formula based on the feature vectors of drugs and proteins. We still used 10 trials of pairwise 5-fold cross-validation and conducted sub-dataset extraction for PU learning, similar to the previous section.

**Figure 6:**
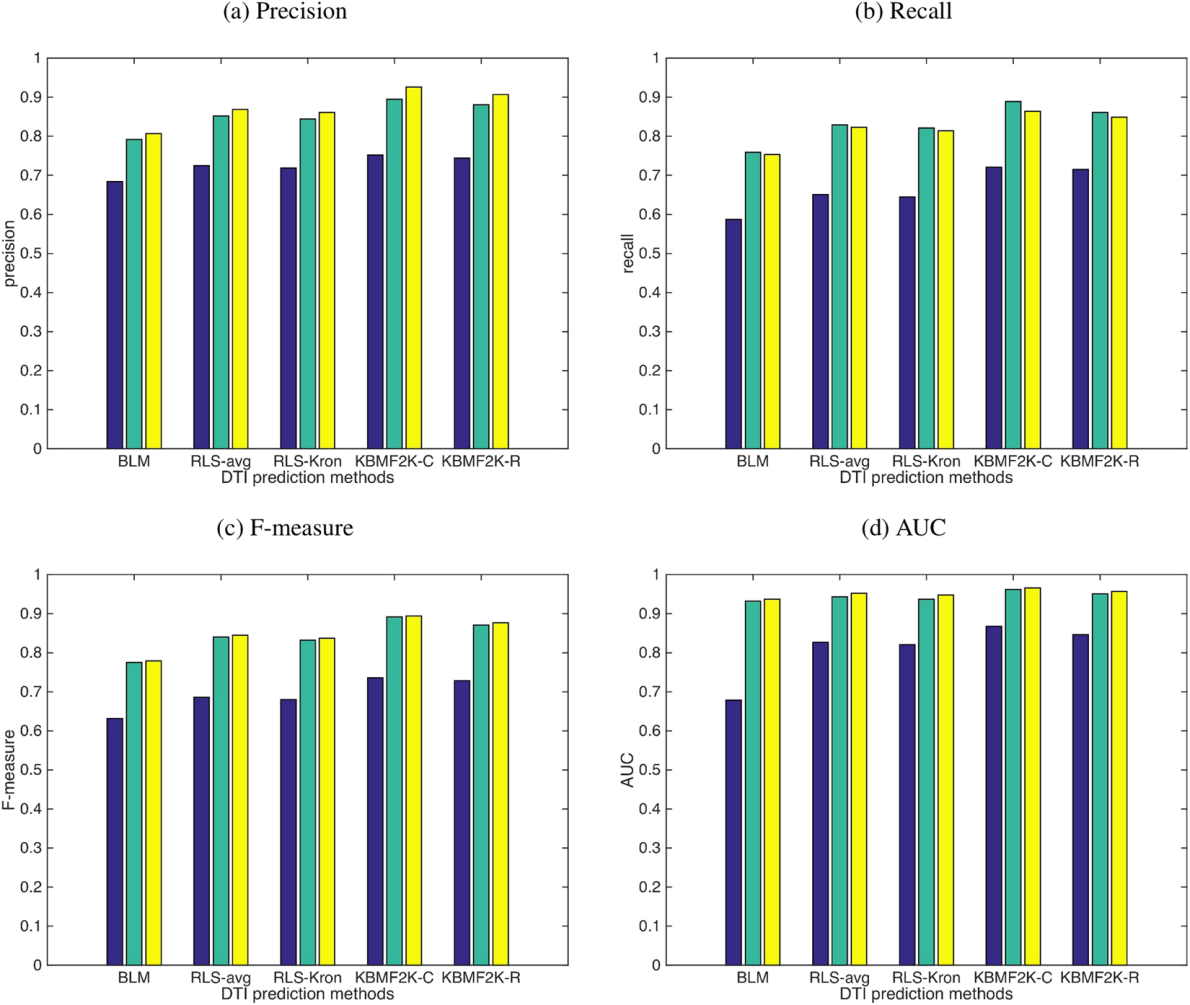
Performance comparison of different negative sample selection methods. Blue denotes the performances of random selection method, green denotes the performances of NCPIS and yellow denotes the performances of our proposed NDTISE method. (**a**–**d**) Represent precision, recall, F-measure and AUC values of different negative samples extraction methods using respective classification models, respectively.

The results are as shown in Fig. 6. NDTISE significantly outperforms random selection method in 5 representative DTI prediction models. The recall values of NDTISE were lower than NCPIS in these models. However, the precision values of NDTISE are better than NCPIS, that is, more correctly predicted DTIs were obtained; although, successfully predicted DTIs were relatively few. Moreover, NDTISE obtained better improvement than NCPIS in terms of F-measure and AUC. These results indicated that our designed NDTISE method can extract NDTISs properly.

### Sensitivity Study on the Parameter

The similarity weights of an ambiguous sample are used to measure the probabilities that the sample belongs to the positive and negative classes. The parameter *α* is used to balance the importance between local and global similarities. To measure the sensitivity of *α* in our proposed PUDTI framework, we conducted a series of extensive experiments to investigate the performance under different settings.

**Figure 7:**
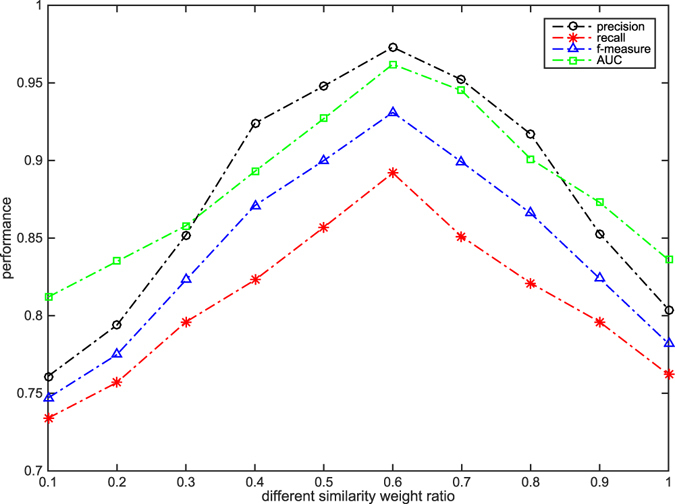
The choice of *α* values.

As described in Fig. 7, when *r* is 30, and if *α* < 0.6, the performances increase gradually; and if *α* > 0.6, the performances decrease gradually. We obtained the similar results when *r* was selected from 40 to 70 with a step size of 10. Therefore, we set *α* as 0.6.

### Drug Repositioning for Astemizole

Astemizole is a long-acting and non-sedative antihistaminic. The drug has antiallergic properties and is used to treat allergic conjunctivitis, asthma, chronic idiopathic urticaria and seasonal allergic rhinitis^36^. Recently, ref. 37 reported that astemizole was possibly a new anti-cancer drug. Therefore, identifying new drug repositioning candidates for the drug is significant. We intended to find new association information for the drug from the DrugBank database^38^ by training SVM-SW classification model after determining the performances of PUDTI.

**Figure 8:**
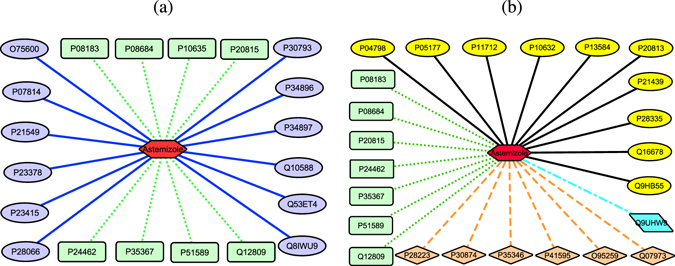
New drug repositioning candidates of astemizole. Figure (**a**) describes the known DTIs and extracted NDTISs of astemizole. Red hexagon denotes astemizole, the green dotted lines denote known DTIs, the blue solid lines denote extracted NDTISs in (**a**). Figure (**b**) describes predicted DTIs of astemizole. The green dotted lines denote successfully predicted DTIs, the orange dash lines denote predicted DTIs that can be validated by the related databases, the azure dash dotted line denotes predicted DTIs which have been reported by ref. 26, the black solid lines denote the other predicted results in (**b**).

Astemizole interacts with eight proteins, namely, P24462, P08183, P35367, P51589, P20815, P10635, P08684 and Q12809 in the DrugBank database^38^. We extracted twelve negative DTIs for the drug, namely, O75600, P07814, P21549, P23378, P23415, P28066, P30793, P34896, P34897, Q10588, Q53ET4 and Q8IWU9. Five of these extracted negative DTIs have been reported by ref. 26. We used cytoscape^39^ to draw DTI networks. Figure 8(a) listed known DTIs in the DrugBank database^38^ and reliable NDTISs extracted by algorithm 1.

We predicted possible interaction partners for astemizole based on known DTIs and extracted NDTISs. The predicted results are shown in Fig. 8(b). These DTIs can be divided into four parts: the first part includes known DTIs in the DrugBank database^38^, wherein seven of eight known DTIs are identified by PUDTI. The second part includes DTI candidates that are unknown in the DrugBank database^38^ but can be validated by retrieving the other databases. Among these DTIs, the interactions between astemizole and four proteins, namely, Q07973, O95259, P28223 and P41595, can be validated by searching the STITCH database^40^, and the interactions between astemizole and two proteins, namely, P35346 and P30874, can be substantiated by retrieving the SuperTarget database^41^.

The third part includes the interaction between astemizole and Q9UHW9, which has been reported by ref. 26. The remaining are from the associations between astemizole and P04798, P05177, P10632, P11712, P13584, P20813, P21439, P28335, Q16678 and Q9HB55.

P08183 is an energy-dependent efflux pump and used to decrease drug accumulation in cells^42^. The protein interacts with astemizole in the DrugBank database^38^. Phosphatidylcholine translocator ABCB4 (P21439) is energy-dependent phospholipid efflux translocator and used to positively regulate biliary lipid secretion. It specifically translocates phosphatidylcholine from canalicular membrane bilayer into hepatocytes. The translocation enables biliary phospholipids to be extracted into the canaliculi lumen and thus protects hepatocytes from the detergent properties of bile salts^42^. Both P08183 and P21439 are multidrug resistance proteins^38^. The function of P21439 is similar to P08183’s^41^. Moreover, sequence similarity and sequence identity between these two proteins are 0.86 and 0.753 in the SuperTarget database, respectively^41^. Therefore, we inferred that P21439 may be new drug repositioning candidates of astemizole based on the predictive accuracy of PUDTI, functional similarity, sequence similarity and sequence identity to known target.

### Drug Repositioning for DNA topoisomerase 2-alpha

DNA topoisomerase 2-alpha (P11388) encoded by the TOP2A gene is used to control topological states of DNA. It is essential for segregating daughter chromosomes during mitosis and meiosis^38^. We intended to find new drug repositioning candidates for the protein from the DrugBank database^38^ by training SVM-SW classification model after determining the performances of PUDTI.

**Figure 9:**
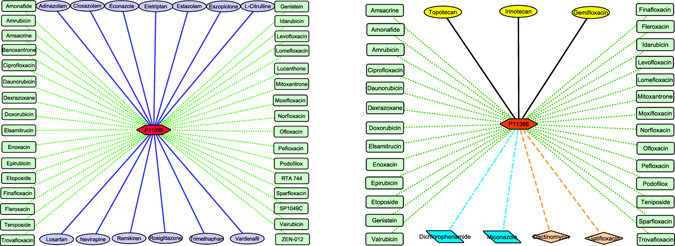
New drug repositioning candidates of P11388. Figure (**a**) describes the known DTIs and extracted NDTISs of P11388. Red hexagon denotes P11388, the green dotted lines denote known DTIs, the blue solid lines denote extracted NDTISs in (**a**). Figure (**b**) describes predicted DTIs of P11388. The green dotted lines denote successfully predicted DTIs, the orange dash lines denote predicted DTIs that can be validated by the related databases, the azure dash dotted lines denote predicted DTIs which have been reported by ref. 26, the black solid lines denote the other predicted results in (**b**).

P11388 interacts with thirty-two drugs in the DrugBank database^38^. Most of these drugs are used to interfere with the transcription process and prevent the RNA synthesis^38^. We extracted thirteen negative DTIs for the proteins, where eight of these extracted negative DTIs have been reported by ref. 26. We used cytoscape^39^ to draw DTI networks. Figure 9(a) listed known DTIs in the DrugBank database^38^ and reliable NDTISs extracted by algorithm 1.

We predicted possible interaction partners for P11388 based on known DTIs and extracted NDTISs. The predicted results were shown in Fig. 9(b). These DTIs can be divided into four parts: the first part includes known DTIs in the DrugBank database^38^, wherein twenty-seven of thirty-two known DTIs are identified by our proposed PUDTI framework. The second part includes DTI candidates that are unknown in the DrugBank database^38^ but can be validated by retrieving the other databases. Among these DTIs, the interaction between dactinomycin and P11388 can be validated by searching the UniProt database^42^, and the interaction between gatifloxacin and P11388 can be substantiated by retrieving the SuperTarget database^41^. Dactinomycin is used to bind to DNA and inhibit RNA synthesis. Protein synthesis, a result of impaired mRNA production, will decline after dactinomycin therapy^38^. Gatifloxacin is used to inhibit bacterial enzymes DNA gyrase. The drug is available in aqueous solutions for intravenous therapy^38^.

The third part includes the interactions between P11388 and dichlorophenamide and miconazole, which have been reported by ref. 26. The remaining are from the associations between P11388 and irinotecan and topotecan. P11388 interacts with camptothecine in the SuperTarget database^41^. Both irinotecan and topotecan are derivatives of camptothecin^38^. Topotecan is a drug used to treat ovarian cancer. It is used to regulate DNA topology and facilitate DNA recombination, replication and repair by inhibiting DNA topoisomerase I^38^. The similarity between camptothecine and topotecan is 0.94 in the SuperTarget database^41^. The association between P11388 and topotecan can be validated by retrieving refs 43–45. Therefore, we inferred that P11388 may interact with topotecan.

### Find New Clues of Treatment for Alzheimer’s Diseases

The above results of drug repositioning imply that existing drugs and drug targets may help find new therapies for diseases. We investigated the complex associations between existing drugs and drug targets of Alzheimer’s disease to infer new clues of treatment for the disease. We retrieved six drugs for Alzheimer’s disease based on its indications in the DrugBank database, namely, galantamine, olanzapine, quetiapine, risperidone, thioridazine and ziprasidone^38^. All the other five drugs except for galantamine target seven proteins, namely, D(1A), D(2) and D3 dopamine receptors (P21728, P14416 and P35462), alpha-1A and alpha-1B adrenergic receptor (P35348 and P35368), 5-hydroxytryptamine receptors (P28223) and potassium voltage-gated channel subfamily H member 2(Q12809)^38^.

**Figure 10:**
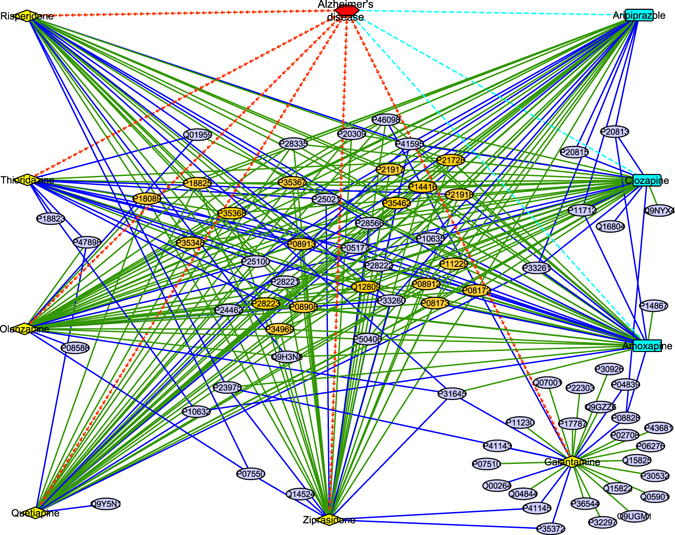
New clues of the treatment of Alzheimer’s disease. Red hexagon denotes Alzheimer’s disease, yellow diamonds denote known drugs of Alzheimer’s disease, azure rectangles denote predicted new clues of treatment of Alzheimer’s disease. Green solid lines denote known DTIs, blue solid lines denote predicted DTIs, red separate arrow lines denote the associations between Alzheimer’s disease and known drugs, azure dash lines denote the associations between Alzheimer’s disease and new clues of treatment.

We found some drugs targeting these seven proteins in the DrugBank database. However, we can not infer new clues of the treatment of Alzheimer’s disease only by these seven target proteins. Therefore, we intended to predict the interactions between these six drugs and targets, as well as the associations between these drug targets and the other drugs. The results are shown in Fig. 10. We can observe that the other five drugs except for galantamine generally target parts of target proteins, namely, adrenergic receptors (P35348, P35368, P08913, P18089 and P18825), dopamine receptors (P21728, P21917, P21918, P35462 and P14416), 5-hydroxytryptamine receptors (P28223, P34969 and P08908), muscarinic acetylcholine receptors (P08172, P08173, P08912 and P11229), histamine H1 receptor (P35367) and potassium voltage-gated channel subfamily H member 2(Q12809). Therefore, we inferred that these target proteins may have a strong correlation with Alzheimer’s disease.

We further considered the other drugs targeting these proteins in the DrugBank database and found that aripiprazole may have strong correlations with these target proteins. Aripiprazole is atypical antipsychotic medication and is used to treat schizophrenia and mediate its antipsychotic effects primarily by P14416. It has been reported in ref. 46 that aripiprazole may be in clinical trails and used to the treatment of Alzheimer’s disease. Therefore, we inferred that aripiprazole may be a drug candidate of Alzheimer’s disease.

## Discussion

Supervised learning-based methods demonstrated better classification performances for potential DTI identification than traditional computational methods. However, experimentally validated NDTISs were impossible to achieve or even unavailable. Therefore, screening negative training samples for DTI prediction models is a recurring problem. In this study, we designed the NDTISE method to extract reliable NDTISs based on PU learning and various biological information. A novel DTI screening framework, PUDTI, is then developed to find new drug repositioning candidates of existing drugs and targets. Experimental results from three different negative sample selection methods on the DTI data provided by NCPIS^26^, 6 state-of-the-art methods on 4 classes of DTI datasets from human nuclear receptors, GPCRs, ion channels and enzymes, and 5 representative DTI prediction models on the DrugBank data demonstrated the generalization capability and competitiveness of our proposed PUDTI framework. The framework identified new drug repositioning candidates for the drug astemizole and the target DNA topoisomerase 2-alpha, and found new clues of the treatment for Alzheimer’s disease.

The PUDTI framework can produce good results over all measures compared with different methods. This observation may be ascribed to the following advantages of the framework. (1) The framework can effectively extract those DTI candidates that are most likely to be negative samples. These NDTISs are applied to identify possible DTIs with the labeled DTIs. (2) The framework took advantage of multiple classifier combination and effectively integrated two types of PU learning models and various biological information related to drugs and targets. (3) In the DTI prediction problem, the noise in training samples was unavoidable. Different similarity weights were calculated to demonstrate different noise levels of the ambiguous samples. Therefore, the built SVM-SW was more tolerant to different noise levels of various DTI data types.

The PUDTI framework integrated the Spy and Rocchio classifiers^32, 33^ to extract reliable NDTISs. However, the predictive accuracy can be further improved by integrating multiple PU learning models. In subsequent investigations, we will consider an ensemble PU learning framework for DTI screening to minimize the possible bias and errors in these two types of PU learning methods.

The negative sample construction is a key issue in predicting associations between various biological entities, such as lncRNA-disease associations, miRNA-disease associations and drug-drug associations. The PUDTI framework may also benefit from the extraction of various negative samples, which will in turn assist in identifying underlying associations between these entities. In further experiments, we will consider to build negative lncRNA-disease association dataset and negative miRNA-disease association dataset based on PU learning to improve predictive performance.

Finding new therapies for existing drugs is significant for modern drug development. There are complex associations between diseases and their known drugs and drug targets. In the future, we will consider to build a supervised learning model by constructing a disease-drug-target network to identify new clues of the treatment for existing diseases.

## Materials and Methods

### Materials

#### Representing Drug Molecules

Different kinds of descriptors were used to describe various drug molecule properties in drug discovery. A PaDEL-Descriptor software^47^ has been designed to represent drug molecules. We used the software and represented a drug molecule as ${\boldsymbol{G}}={({g}_{1},{g}_{2},\ldots ,{g}_{1444})}^{T}$ based on the preprocessing program provided by ref. 25.

#### Representing Target Proteins

Various types of protein descriptors were defined based on different properties of target proteins in proteomics. For representing target proteins, we used three types of protein properties, namely, protein domain^48^, pseudo amino acid composition (PAAC)^49^ and position specific scores^50^.

Protein Domain: Domains of target proteins were retrieved from the PFAM database^48^. A total of 1331 functionally assigned domains on human are available in PFAM. The domain component of a target protein is denoted as ${\boldsymbol{O}}={({o}_{1},{o}_{2},\ldots ,{o}_{1331})}^{T}$, where *o*_*i*_ (1 ≤ *i* ≤ 1331) is equal to 1 if the target protein contains the *i*th domain; otherwise, *o*_*i*_ is equal to 0.

PAAC: The PAAC method^49^ described each protein based on the amino acid sequence of a protein. Following the PAAC method, we used PAAC features as descriptors to represent each target protein as a 50-dimensional vector:

1 $${\boldsymbol{A}}={({a}_{1},{a}_{2},\ldots ,{a}_{20+\lambda })}^{T}\quad (\lambda =30)$$

Position Specific Score Matrix (PSSM): The bi-gram feature extraction method (BiGFE)^51^ was developed to describe the evolutionary information of target proteins combining position specific scoring matrix (PSSM)^50^ of target proteins. References 12 and 52 used the method and obtained improved performances in predicting DTIs. We described each protein as a 400-dimensional feature vector based on the BiGFE method:

2 $${\boldsymbol{B}}={({B}_{1,1},{B}_{1,2},\ldots ,{B}_{i,j},\ldots {B}_{20,1},\ldots ,{B}_{20,20})}^{T}\quad (1\le i\le 20,1\le j\le 20)$$

Combing domains, PAACs and PSSM, a protein target can be represented as a 1781-dimensional vector:

3 $${Q}=[\begin{array}{c}{O}\\ {A}\\ {B}\end{array}]$$

Therefore, each DTI sample can be described as a 3225-dimension vector based on PaDEL-Descriptors of drugs and domains, PAACs and PSSM of target proteins:

4 $${F}=[\begin{array}{c}{G}\\ {Q}\end{array}]$$

${\boldsymbol{F}}={({f}_{1},{f}_{2},\ldots ,{f}_{1444},\ldots ,{f}_{3225})}^{T}$, where $\{{f}_{1},{f}_{2},\ldots ,{f}_{1444}\}$ represents the 1444 PaDEL-Descriptors of drugs, and $\{{f}_{1445},{f}_{1446},\ldots ,{f}_{3225}\}$ represents the 1781 descriptors of target proteins.

#### Drug-target Interaction Data

We downloaded DTI data from STITCH^40^, DrugBank^38^ and Matador^41^, which were provided by ref. 26. In these databases, a total of 2,290,630 interactions between 367,142 unique drug compounds and 19, 342 target proteins on human are available.

### Methods

The proposed PUDTI framework can be divided into five steps:

- Select the feature subsets of DTI samples.
- Screen the high-quality NDTISs.
- Calculate the representative positive and negative prototypes.
- Compute the similarity weights of the ambiguous samples.
- Construct the final classification model and identify DTI candidates.

In the following, we described every step in details.

#### Step 1: Feature Selection

There are parts of robust features in DTI feature set. Selecting a feature subset from these features may help decrease the false positive and the false negative ratios, thereby avoiding the overfitting problem. Reference 53 developed a feature selection method to distinguish disease genes from non-disease genes, we used the method to select feature subsets for each DTI to efficiently distinguish interacting drug-target pairs from noninteracting drug-target pairs.

For each DTI feature *f*, we define its association score in *P* and *U* (*as*(*f*, *P*) and *as*(*f*, *U*)) as follows:

5 $$\begin{array}{l}as(f,P)=\sum _{DT{P}_{i}\in P}\,asso(DT{P}_{i},f)\\ as(f,U)=\sum _{DT{P}_{i}\in U}\,asso(DT{P}_{i},f)\end{array}$$

where *DTP*_*i*_ is the *i*th Drug-Target pair, *DTP*_*i*_ ∈ *P* indicates that the *i*th DTP is positive and *DTP*_*i*_ ∈ *U* represents that the *i*th DTP is unlabeled. *asso*(*DTP*_*i*_, *f*) represents the association score between *DTP*_*i*_ and the feature *f*, which can be computed as follows:

6 $$asso(DT{P}_{i},f)=\{\begin{array}{l}1\quad if\,DT{P}_{i}\,have\,feature\,f\\ 0\quad if\,DT{P}_{i}\,have\,not\,feature\,f\end{array}$$

We then compute the discriminant ability score of *f* in *P* and *U* as,

7 $$da(f)=(as(f,P)+as(f,U))\ast \mathrm{log}(\frac{|P|}{as(f,P)}+\frac{|U|}{as(f,U)})$$

By Eq. (7), we intend to screen those discriminative features which either frequently present in *P* but seldom in *U* or frequently present in *U* but seldom in *P*. For a feature *f*, when *as*(*f*, *P*) in *P* is large but *as*(*f*, *U*) in *U* is small or *as*(*f*, *U*) in *U* is large but *as*(*f*, *P*) in *P* is small, *da*(*f*) will be large because both *af*(*f*, *P*) + *af*(*f*, *U*) and *log*(|*P*|/*af*(*f*, *P*) + |*U*|/*af*(*f*, *U*)) are relatively large. On the contrary, the score will be relatively low when both *af*(*f*, *P*) and *af*(*f*, *U*) are small or large simultaneously. Thus, we can select representative feature subsets for each DTI.

#### Step 2: Screening Reliable NDTISs

Typically, supervised learning-based models require numerous labeled positive and negative samples to achieve good classification accuracy. However, known DTIs are rare, and NDTISs are difficult to achieve or even unavailable. Moreover, numerous DTI examples are unlabeled. To obtain a good predictive performance, we intend to screen trustworthy NDTISs.

We considered two classical PU learning models, namely, the Spy and Rocchio techniques^32, 33^. To reduce the expected error rates when screening NDTISs, we minimized the bias of individual model based on multiple classifier combination. The details are described in algorithm 1.

In algorithm 1, *RN* and *EP* denote reliable NDTISs and positive samples extracted by algorithm 1, respectively. *C*_*Spy*_ and *C*_*Roc*_ represent the classification results from the Spy and Rocchio classifiers^32, 33^, respectively. Steps 1 and 2 initialize *P*, *U*, *RN* and *EP*. Steps 3–5 classify the unknown DTIs in *U*. Steps 6–9 screen *RN* by excluding positive DTIs as far as possible. For instance, a DTI is regarded as a reliable negative sample if its classification results from two classifiers are both negative classes, that is, the DTI simultaneously satisfies *C*_*Spy*_ = −1 and *C*_*Roc*_ = −1. Steps 10–14 are used to add high-quality positive examples to *P*. The *U* in Step 15 denotes the remaining unlabeled DTIs after extracting parts of high-quality positive and negative examples. We considered these remaining DTIs as the ambiguous samples.

**Algorithm 1:**
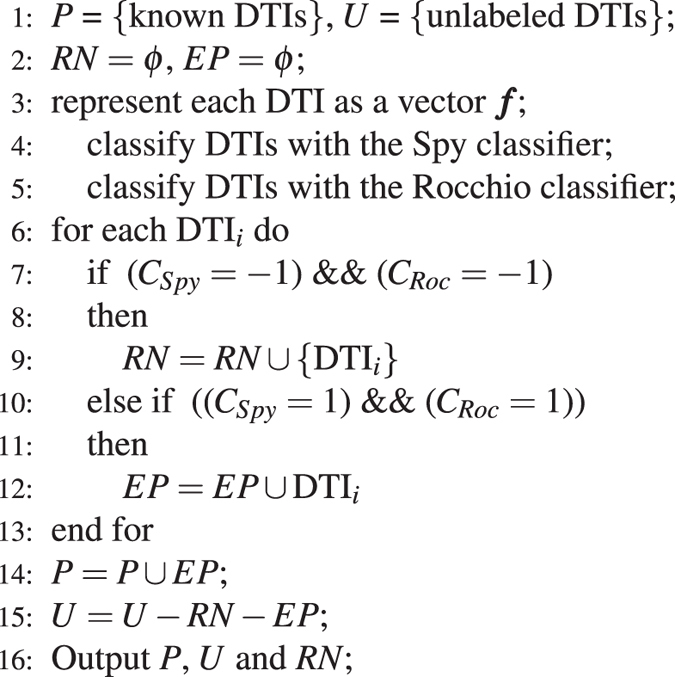
The NDTISE method.

#### Step 3: Computing the Representative Positive and Negative DTI Prototypes

We achieved reliable NDTISs from the last section. In theory, we can build a classifier and predict new DTIs using *P* and *RN*. However, the classification results may not be accurate enough because parts of ambiguous samples remain. For these ambiguous samples, we cannot determine whether they belong to the positive or negative classes. Assigning these examples to the positive or negative class will disturb the classification performance. As such, considering the method provided by refs 29 and 31, we developed a similarity weight calculation method to measure the probabilities that remaining ambiguous samples belong to the positive and negative classes.

To compute the similarity weights of these ambiguous samples, we partitioned DTI samples in *RN* into *a* modules using the *k*-means clustering algorithm and computed the representative positive and negative DTI prototypes. The details are described in algorithm 2.

**Algorithm 2:**
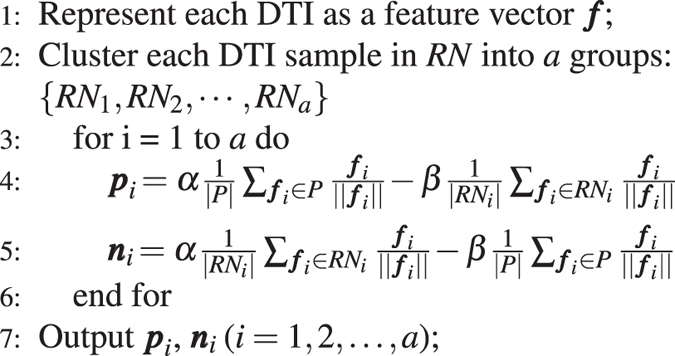
Computing the representative positive and negative DTI prototypes.

The parameter *a* was set as $a=t\ast |RN|/(|U|+|RN|)$, where |*RN*| and |*U*| denote the numbers of *RN* and *U*, respectively. *t*, *α* and *β* were set as 30, 16 and 4, respectively, as recommended by the studies^29–31^.

#### Step 4: Computing the Similarity Weights of the Ambiguous Samples

The similarity weights of the remaining ambiguous samples in *U* represent the probabilities that the samples belong to the positive and negative DTI classes. To compute the similarity weights, we defined the similarities of an ambiguous sample *x* to the *i*th representative positive and negative prototypes (*p*_*i*_ and *n*_*i*_) as follows:

8 $$\begin{array}{l}sim({\boldsymbol{x}},{{\boldsymbol{p}}}_{i})=\frac{{\boldsymbol{x}}\cdot {{\boldsymbol{p}}}_{i}}{\Vert {\boldsymbol{x}}\Vert \cdot \Vert {{\boldsymbol{p}}}_{i}\Vert }\\ sim({\boldsymbol{x}},{{\boldsymbol{n}}}_{i})=\frac{{\boldsymbol{x}}\cdot {{\boldsymbol{n}}}_{i}}{\Vert {\boldsymbol{x}}\Vert \cdot \Vert {{\boldsymbol{n}}}_{i}\Vert }\end{array}$$

Computing Local Similarity Weights: We developed an algorithm to measure the local similarity weights of the ambiguous samples.

**Algorithm 3:**
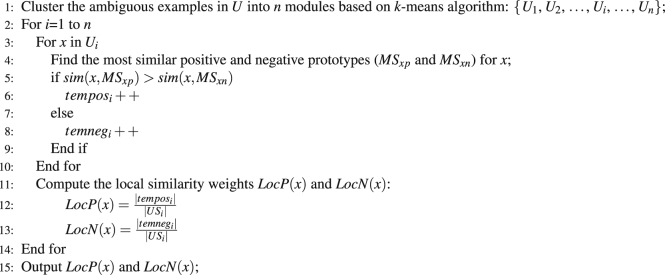
Computing the Local similarity weights of the ambiguous samples.

where *n* is set as $n=t\ast |U|/(|U|+|RN|)$ and *t* is set as 30, which are recommended by refs 29 and 31. Step 5–9 tag *x* with a temporary label. |*US*_*i*_| denotes the number of all samples in *US*_*i*_. |*tempos*_*i*_| denotes the number of samples which are temporarily regarded as positive samples in *US*_*i*_, |*temneg*_*i*_| denotes the number of samples which are temporarily regarded as negative samples in *US*_*i*_. The most similar positive and negative prototypes of *x* can be obtained by equation (8).

**Figure 11:**
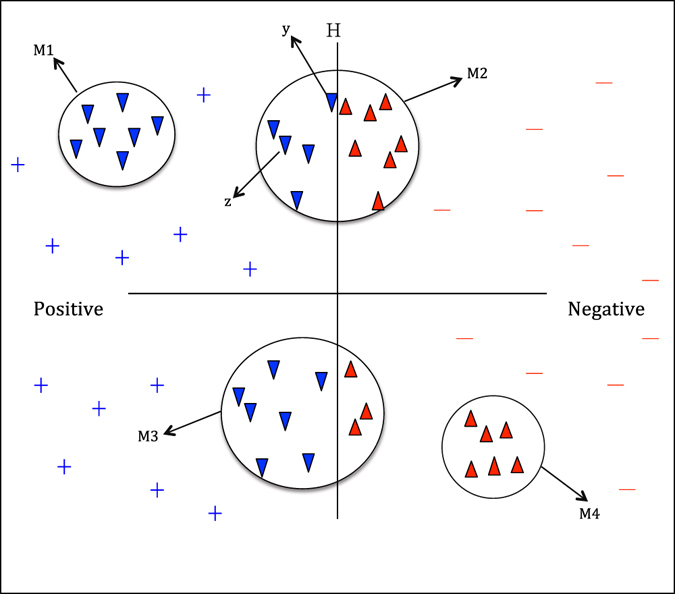
Computing the local similarity weights of the ambiguous samples. Blue lower triangles represent positive DTI samples in a cluster, red upper triangles represent NDTISs in the cluster.

As illustrated in Fig. 11, *H* denotes the decision hyperplane in the process of classification and can be computed by the Rocchio classifier^33^. The ambiguous examples in *U* are clustered into four modules, namely, *M*_1_, *M*_2_, *M*_3_ and *M*_4_. The examples in *M*_1_, *M*_2_, *M*_3_ and *M*_4_ are assigned with local similarity weights (1, 0), $(\tfrac{5}{12},\tfrac{7}{12})$, $(\tfrac{7}{10},\tfrac{3}{10})$ and (0, 1), respectively.

Computing Global Similarity Weights: The local similarity weights utilized the biological features shared by the ambiguous samples and computed the similarities between all samples in a cluster. However, the local similarity weights of samples in the same cluster are possibly different because of different physical locations. For example, assigning the same class weight to the ambiguous samples ***y*** and ***z*** in *M*_2_ is inappropriate even though the two samples have the same local similarity weights. Therefore, we calculated the global similarity weights between *x* and all representative prototypes to measure the probabilities that *x* belongs to the positive and negative DTI classes from a global perspective.

The global similarity weights of ***x*** can be measured as follows:

9 $$\begin{array}{l}GloP({\boldsymbol{x}})=\frac{{\sum }_{i=1}^{a}sim({\boldsymbol{x}},{{\boldsymbol{p}}}_{i})}{{\sum }_{i=1}^{a}(sim({\boldsymbol{x}},{{\boldsymbol{p}}}_{i})+sim({\boldsymbol{x}},{{\boldsymbol{n}}}_{i}))}\\ GloN({\boldsymbol{x}})=\frac{{\sum }_{i=1}^{a}sim({\boldsymbol{x}},{{\boldsymbol{n}}}_{i})}{{\sum }_{i=1}^{a}(sim({\boldsymbol{x}},{{\boldsymbol{p}}}_{i})+sim({\boldsymbol{x}},{{\boldsymbol{n}}}_{i}))}\end{array}$$

where *GloP*(***x***) and *GloN*(***x***) represent the probabilities that *x* belongs to the positive and negative DTI classes from a global perspective.

We obtain the final probabilities that ***x*** belongs to the positive and negative DTI classes based on its local and global similarity weights:

10 $$\begin{array}{l}{W}^{P}({\boldsymbol{x}})=(1-\alpha )LocP({\boldsymbol{x}})+\alpha GloP({\boldsymbol{x}})\\ {W}^{N}({\boldsymbol{x}})=\mathrm{(1}-\alpha )LocN({\boldsymbol{x}})+\alpha GloN({\boldsymbol{x}})\end{array}$$

where the parameter *α* is used to balance the importance between the global similarity and the local similarity.

#### Step 5: Constructing SVM-based Classification Model

By incorporating positive DTI dataset *P*, reliable negative DTI dataset *RN*, the similarity weights of the ambiguous examples in *U*, we obtained training datasets to learn classification model for novel DTI identification. These training examples may include parts of noisy data. Therefore, we built an SVM with similarity weights (SVM-SW) as our basic classifier to tolerate these noisy examples.

Constructing Classification Model: SVM^54^ is a powerful tool for data classification. We classified unknown DTIs based on SVM. Suppose that

$X=\{({x}_{1},{y}_{1}),({x}_{2},{y}_{2}),\ldots ,({x}_{n},{y}_{n})\}$ be training dataset. *x*_*i*_ denotes the *i*th DTI sample and can be represented as a feature vector ***x***_*i*_ after feature selection in Step 1, *y*_*i*_ ∈ {+1, −1}. We can classify the unknown DTIs based on standard SVM:

11 $$\begin{array}{l}\mathop{{\rm{\min }}}\limits_{{\boldsymbol{w}},b,\varepsilon }\frac{1}{2}{\Vert {\boldsymbol{w}}\Vert }_{2}^{2}+C\sum _{{x}_{i}\in P\cup RN\cup U}\,{\varepsilon }_{i}\\ \begin{array}{ll}s.t. & {y}_{i}({{\boldsymbol{w}}}^{T}\,{{\boldsymbol{x}}}_{{\boldsymbol{i}}}+b)\ge 1-{\varepsilon }_{i},\,{x}_{i}\in P\cup RN\cup U\\  & {\varepsilon }_{i}\ge 0,{x}_{i}\in P\cup RN\cup U\end{array}\end{array}$$

where *ε*_*i*_ is a slack variable of *x*_*i*_ and is used to allow for misclassifications in the training examples, and *C* is used to balance the impact of *ε*_*i*_. The test sample *x* is viewed as the positive class if ***w*** · *ϕ*(***x***) + *b* > 0; otherwise, it is negative.

Combining standard SVM with the similarity weights of the ambiguous samples, we further introduced SVM-SW for finding DTI candidates:

12 $$\begin{array}{l}{\rm{\min }}\,F({\boldsymbol{w}},b,\varepsilon )=\frac{1}{2}{\Vert {\boldsymbol{w}}\Vert }^{2}+{C}_{1}\sum _{i=1}^{|P|}\,{\varepsilon }_{i}+{C}_{2}\sum _{j=1}^{|U|}\,{W}^{P}({{\boldsymbol{x}}}_{j}){\varepsilon }_{j}+{C}_{3}\sum _{m=1}^{|U|}\,{W}^{N}({{\boldsymbol{x}}}_{m}){\varepsilon }_{m}+{C}_{4}\sum _{n=1}^{|RN|}\,{\varepsilon }_{n}\\ \begin{array}{ll}s.t. & {y}^{(i)}({{\boldsymbol{w}}}^{T}\,{{\boldsymbol{x}}}^{(i)}+b)\ge 1-{\varepsilon }_{i},{{\boldsymbol{x}}}^{(i)}\in P\\  & {y}^{(j)}({{\boldsymbol{w}}}^{T}\,{{\boldsymbol{x}}}^{(j)}+b)\ge 1-{\varepsilon }_{j},{{\boldsymbol{x}}}^{(j)}\in U\\  & {y}^{(m)}({{\boldsymbol{w}}}^{T}\,{{\boldsymbol{x}}}^{(m)}+b)\le -1+{\varepsilon }_{m},{{\boldsymbol{x}}}^{(m)}\in U\\  & {y}^{(n)}({{\boldsymbol{w}}}^{T}\,{{\boldsymbol{x}}}^{(n)}+b)\le -1+{\varepsilon }_{n},{{\boldsymbol{x}}}^{(n)}\in RN\\  & {\varepsilon }_{i}\ge 0,\,\,{\varepsilon }_{j}\ge \mathrm{0,}\,\,{\varepsilon }_{m}\ge \mathrm{0,}\,\,{\varepsilon }_{n}\ge 0\end{array}\end{array}$$

where *ε*_*i*_, *ε*_*j*_, *ε*_*m*_ and *ε*_*n*_ are the error terms. *C*_1_, *C*_2_, *C*_3_ and *C*_4_ are penalty factors that are used to control the trade-off between margin and misclassification errors. *W* ^*P*^(***x***_*j*_)*ε*_*j*_ and *W* ^*N*^(***x***_*m*_)*ε*_*m*_ are errors with different weights. Different *W* ^*P*^(***x***_*j*_) and *W* ^*N*^(***x***_*m*_) reflect different effects of the parameters *ε*_*j*_ and *ε*_*m*_ on classification accuracy, respectively. The large value of *W* ^*P*^(***x***_*j*_) can increase the effect of *ε*_*j*_; therefore, the ambiguous example ***x***_*j*_ is more likely to belong to the positive class. Similarly, the smaller value of *W* ^*N*^(***x***_*m*_) can reduce the effect of *ε*_*m*_; therefore, ***x***_*m*_ is less significant toward the negative class.

Solving the Model: The model can be solved based on the method provided by refs 29 and 31. For a test sample *x*, it is regarded as a positive DTI if ***w*** · *ϕ*(***x***) + *b* > 0; otherwise, it is regarded as a negative DTI.

### Experimental Setup and Evaluation Metrics

Various performance measures have been proposed to evaluate DTI prediction models. Among these, precision, recall, AUC and F-measure are extensively used. Precision, recall and F-measure^26^ are computed as equations (13)–(15):

13 $$Precision=\frac{TP}{TP+FP}$$

14 $$Recall=\frac{TP}{TP+FN}$$

15 $$F-measure=\frac{2\ast Precision\ast Recall}{Precision+Recall}$$

where *TP*, *FP*, *TN* and *FN* represent true positive, false positive, true negative, and false negative, respectively.

Precision is the percentage of correctly predicted DTIs and is used to measure the distinguished capability of a classifier. Recall is the percentage of successfully predicted DTIs. F-measure is used to evaluate the average classification performance. Either small precision or recall will result in a low F-measure^30^: therefore, F-measure is used to measure predictive models. AUC is the average area under the receiver operating curve. For these four parameters, higher values exhibit better classification performance. We used these four metrics to evaluate our proposed PUDTI framework.
